# Correlation of caecal microbiome endotoxins genes and intestinal immune cells in *Eimeria tenella* infection based on bioinformatics

**DOI:** 10.3389/fcimb.2024.1382160

**Published:** 2024-03-20

**Authors:** Mingzheng Han, Jiale Li, Yijin Wu, Jianzhao Liao

**Affiliations:** ^1^ College of Veterinary Medicine, South China Agricultural University, Guangzhou, China; ^2^ Department of Blood Transfusion, Yuexi Hospital of the Sixth Affiliated Hospital, Sun Yat-sen University (Xinyi People’s Hospital), Xinyi, China

**Keywords:** Eimeria tenella, intestinal immune cells, cecal microbial, endotoxins, GEO datasets

## Abstract

**Introduction:**

The infection with *Eimeria tenella* (ET) can elicit expression of various intestinal immune cells, incite inflammation, disrupt intestinal homeostasis, and facilitate co-infection with diverse bacteria. However, the reciprocal interaction between intestinal immune cells and intestinal flora in the progression of ET-infection remains unclear.

**Objective:**

The aim of this study was to investigate the correlation between cecal microbial endotoxin (CME)-related genes and intestinal immunity in ET-infection, with subsequent identification of hub potential biomarker and immunotherapy target.

**Methods:**

Differential expression genes (DEGs) within ET-infection and hub genes related to CME were identified through GSE39602 dataset based on bioinformatic methods and Protein-protein interaction (PPI) network analysis. Moreover, immune infiltration was analyzed by CIBERSORT method. Subsequently, comprehensive functional enrichment analyses employing Kyoto Encyclopedia of Genes and Genomes (KEGG) pathway analysis along with Gene Ontology (GO), gene set enrichment analysis (GSEA), and gene set variation analysis (GSVA) were performed.

**Results:**

A total of 1089 DEGs and 25 hub genes were identified and CXCR4 was ultimately identified as a essential CME related potential biomarker and immunotherapy target in the ET-infection. Furthermore, activated natural killer cells, M0 macrophages, M2 macrophages, and T regulatory cells were identified as expressed intestinal immune cells. The functional enrichment analysis revealed that both DEGs and hub genes were significantly enriched in immune-related signaling pathways.

**Conclusion:**

CXCR4 was identified as a pivotal CME-related potential biomarker and immunotherapy target for expression of intestinal immune cells during ET-infection. These findings have significant implications in elucidating the intricate interplay among ET-infection, CME, and intestinal immunity.

## 1 Introduction

Coccidiosis, an acute epidemic intestinal disease of poultry, is caused by the parasitic protozoan genus *Eimeria*. Among these species, *Eimeria tenella* (ET) exhibits the highest pathogenicity (Blake et al., 2015). The proliferation of ET in cecal epithelial cells induces acute inflammation, while concurrently promoting local recruitment of T cells, natural killer (NK) cells, and macrophages (Lillehoj and Trout, 1996). During ET-infection, cecal tissue exhibited increased levels of various interleukins (IL), interferon (IFN), transforming growth factor β (TGF-β) 1-4, tumor necrosis factor α (TNF-α), and TNF superfamily 15 (TNFSF15) (Laurent et al., 2001; Hong et al., 2006; Kim et al., 2019a). Immunity plays a pivotal role in the pathogenesis of ET-infection, and Nod-like receptors (NLRs) have been demonstrated to be associated with diverse protozoan infections (Gurung and Kanneganti, 2016). The IFN-γ-mediated T helper (Th) 1 cells response was initially considered as the primary immune response to coccidiosis, while Th17 and T regulatory (Treg) cells also play a crucial role in maintaining intestinal homeostasis (Kim et al., 2019a). However, the correlation between intestinal immune cells and ET-infection remains unclear.

The cecum harbors the highest abundance and diversity of bacteria within the gastrointestinal tract, and alterations in both microbial diversity and composition have been observed as a consequence of ET-infection (Wei et al., 2013; Sergeant et al., 2014; Qi et al., 2019). Additionally, coccidia infection induces an increase in intestinal permeability, often resulting in secondary bacterial infection (Pham et al., 2021). Consequently, this leads to dysbiosis of the intestinal microbiota and exacerbates infections caused by *Clostridium perfringens*, *Salmonella enterica serovars* Enteritis, or Typhimurium (Arakawa et al., 1981; Qin et al., 1995; Moore, 2016). The alteration of intestinal microflora can promote the proliferation and infection of ET, primarily attributed to the facilitation of acute inflammatory response by microflora (Gaboriaud et al., 2020; Tomal et al., 2023a). The presence of endotoxins can activate macrophages and neutrophils, thereby concurrently inducing the expression of TNF-α and IL-1. Among these cytokines, the upregulation of TNF-α may plays a pivotal role in facilitating ET-infection (Zhang et al., 1995; Stephens and Mythen, 2000). Consequently, genes associated with caecal microbiome endotoxins (CME) possess the potential to modulate ET-infection by exerting influence on host immunity. The infection of ET generally leads to expression of intestinal immune cells and disruption of the cecal flora balance, thereby facilitating secondary bacterial and pathogen infections. However, the intricate interplay between cecal flora, immunity, and ET-infection remains poorly elucidated, with the core genes involved yet to be identified.

Weighted gene co-expression network analysis (WGCNA) is a systems biology method employed to characterize patterns of gene associations across different samples. It can be utilized for the identification of gene sets exhibiting high covariation and for pinpointing potential biomarker genes or therapeutic targets based on the endogeneity of the gene set and its association with the phenotype (Lin et al., 2021). Instead of solely focusing on DEGs, WGCNA leverages information from thousands or nearly 10,000 of the most diverse genes or all genes to identify gene sets of interest and conduct significant association analysis with phenotypes. The primary objective is to maximize the utilization of available information, while the secondary goal is to transform the associations between numerous genes and phenotypes into associations between a select few gene sets and phenotypes, thereby addressing the issue of multiple hypothesis testing and correction (Nangraj et al., 2020). Least Absolute Shrinkage and Selection Operator (LASSO) is a linear regression technique that utilizes L1 regularization. By incorporating L1 regularization, specific learned features are assigned zero weights, thereby achieving the objective of sparsity and feature selection. The fundamental concept underlying the Lasso method is to minimize the sum of squared residuals while constraining the absolute sum of regression coefficients below a constant value. This enables the generation of precisely equal-to-zero regression coefficients and facilitates obtaining an interpretable model (Tibshirani, 1996). By utilizing the WGCNA and LASSO algorithms, we have gained valuable insights into the fundamental modules and biomarkers associated with gene expression, thereby enhancing the scientific rigor of our study.

In this study, the GSE39602 dataset was obtained from the GEO database. The Linear models for microarray data (LIMMA), WGCNA, and LASSO algorithms and Protein-protein interaction (PPI) were employed to identify differentially expressed genes (DEGs) and hub genes, while the CIBERSORT algorithms were utilized for the screening of activated NK cells, M0 macrophages, M2 macrophages, and Treg cells which were expressed in ET-infection. The functional enrichment analysis revealed a significant enrichment of both DEGs and hub genes in immune-related signaling pathways. The core potential biomarker and immunotherapy target of CME associated with the expression of four intestinal immune cells in ET-infection was ultimately identified as CXCR4, providing valuable references into the relationship and functionality of CME-related genes with intestinal immune cells during ET-infection.

## 2 Materials and methods

### 2.1 Acquisition of data sources and identification of differentially expressed genes (DEGs)

The related ET-infection datasets were obtained from the NCBI GEO common repository (http://www.ncbi.nlm.nih.gov/geo) (Barrett et al., 2013) by employing the search term “coccidiosis”. Further refinement was carried out based on sequencing type (array analysis), animal species (Gallus gallus), and sample source (tissue). GSE39602 was generated utilizing the GPL3213 platform and consisted of three ET-infected cecum samples along with three control cecum samples. The genes associated with coccidiosis were retrieved from the CTD database (https://ctdbase.org/) (Davis et al., 2023) using “coccidiosis” as the search query. Furthermore, the genes related to endotoxins were obtained from the CTD database by employing the search term “endotoxins”.

LIMMA is a differential expression screening approach based on generalized linear models (Ritchie et al., 2015). In this study, we utilized the R software package limma (version 3.40.6) to perform differential analysis and identify genes that exhibit differential expression between various comparison groups and control groups. Specifically, after obtaining the expression profile dataset, we initially applied log2 transformation to the data and then conducted multiple linear regression using the lmFit function. Subsequently, we employed the eBays function to compute moderated t-statistics, moderated F-statistics, and log-odds of differential expression through empirical Bayes moderation of standard errors towards a common value. The identification of DEGs was accomplished through a meticulous investigation employing the limma test, which necessitated a stringent p value threshold of 0.05 and a log2 fold change ≥1.

### 2.2 WGCNA and module identification

Modules were obtained by WGCNA using the expression matrix of GSE39602. Initially, Pearson’s correlation matrices and average linkage method were applied to all pairwise genes. Then, a weighted adjacency matrix was created using a power function: A_mn=|C_mn|^β (where C_mn represents Pearson’s correlation between Gene_m and Gene_n; A_mn denotes adjacency between Gene m and Gene n). The parameter β served as a soft-thresholding parameter that emphasized strong correlations while penalizing weak ones. After selecting a power value of 9, the adjacency matrix was transformed into a topological overlap matrix (TOM), which measured the network connectivity of each gene by summing its adjacencies with all other genes in the network generation. The corresponding dissimilarity (1-TOM) was calculated accordingly. To classify genes with similar expression profiles into modules, average linkage hierarchical clustering based on TOM-based dissimilarity measure was performed with a minimum module size of 30 for gene groups in dendrogram analysis while maintaining sensitivity level at three during this process. Furthermore,dissimilarity for module eigenGenes was computed to analyze modules in more detail,and an appropriate cut line for merging certain modules was determined. Finally, the hub modules and module-hub genes are obtained.

### 2.3 Functional enrichment analysis of biological variables

The Kyoto Encyclopedia of Genes and Genomes (KEGG) rest API (https://www.kegg.jp/kegg/rest/keggapi.html) is utilized for gene set function analysis enrichment, obtaining the latest KEGG pathway gene annotation as the background to map genes in the collection. Enrichment analysis was conducted using clusterProfiler package (version 3.14.3) in R software to obtain gene set enrichment results. A minimum of 5 and a maximum of 5000 genes were considered for each gene set, with a P value threshold of <0.05 and a false discovery rate (FDR) threshold of <0.25, indicating statistical significance.

For the functional enrichment analysis of gene sets, we utilized the Gene Ontology (GO) annotation from the R software package org.Hs.eg.db (version 3.1.0) as the background to map genes into the reference set. Subsequently, we performed enrichment analysis using clusterProfiler (version 3.14.3), an R software package. The minimum gene set size was set to 5, while the maximum gene set size was limited to 5000. Statistical significance was determined by a P-value threshold of <0.05 and FDR below <0.25.

Gene Set Enrichment Analysis (GSEA) is employed to assess the distribution pattern of genes within a predefined gene set in a ranked gene list based on their phenotypic relevance, facilitating the evaluation of their contribution to the phenotype. For GSEA, we performed the analysis using GSEA software (version 3.0) obtained from the GSEA website (http://software.broadinstitute.org/gsea/index.jsp) (Subramanian et al., 2005). The sample was stratified into two groups based on Comment information for GSE39602, and Molecular Signatures Database (http://www.gsea-msigdb.org/gsea/downloads.jsp) (Liberzon et al., 2011) was utilized to download the c5.Go.Bp.V7.4.Symbols GMT collections for gene expression profile and phenotypic grouping analysis. We set a minimum gene set size of 5 and a maximum gene set size of 5000, performed 1000 resampling iterations, considered P value <0.05 and FDR <0.25 as statistically significant requirement.

The Gene Set Variation Analysis (GSVA) is an algorithm employed in the GSEA, facilitating unsupervised classification of samples based on alterations in pathway activity by incorporating gene expression and multiple pathway information from the outset. The R packages from GSVA (version 1.40.1) (Hanzelmann et al., 2013) were employed for GSVA. To compute the enrichment score of each sample in the gene set, we utilized Hanzelmann et al’s method (Hanzelmann et al., 2013) and Molecular Signatures Database (http://www.gsea-msigdb.org/gsea/downloads.jsp) (Liberzon et al., 2011) to download the c5.Go.V7.4.Symbols.GMT collections for predefining the gene rank. A minimum gene set size of 5 and a maximum gene set size of 5000 were defined to assess relevant pathways and molecular mechanisms, followed by calculation of the enrichment score for each sample in each gene set. Finally, an enrichment score matrix was obtained.

### 2.4 Acquisition of hub genes and Lasso analysis

The intersection of DEGs, coccidiosis-related genes, CME related genes and module-hub genes was obtained by Venny 2.1.0, namely hub genes. Moreover, we employed the R software package glmnet to integrate survival time, survival state, and hub genes expression data and performed regression analysis using the lasso-cox method. Furthermore, we implemented a 3-fold cross-validation approach to obtain the optimal model. The Lambda value was set at 0.012. The area under curve (AUC) was calculated using the R software package pROC (version 1.17.0.1) through Receiver operating characteristic (ROC) analysis.

### 2.5 Construction of Protein-Protein interaction (PPI) network

The information on the protein interaction network for DEGs and hub genes was obtained from the STRING database (https://cn.string-db.org/) (Szklarczyk et al., 2016), respectively. The screening criteria for organisms are set to “Gallus gallus,” with a minimum required interaction score of “medium confidence (0.4)”. PPI information was imported into Cytoscape 3.10.1 for visualization. To identify hub targets, three Cytoscape plug-ins were integrated for data analysis, with the specific coefficients outlined as follows. According to the Centiscape 2.2 plug-in, betweenness centrality (BC), closeness centrality (CC), and degree values were utilized for an in-depth analysis of node properties within the interaction network. Target nodes were selected based on their degree values, BC, and CC, which exceeded the corresponding median values observed in the PPI network. According to the MCODE plug-in, strongly connected regions were identified using the following cut-off values: degree cutoff = 2, node score cutoff = 0.2, k-core = 2, and maximum depth = 100. The CytoHubba plug-in enables the identification of top10 node genes in ten different ways, allowing for the selection of key gene intersections.

### 2.6 Identification and correlation of immune infiltrating cells in ET-infection

The CIBERSORT algorithm employs linear support vector regression to deconvolute gene expression profiles, utilizing RNA sequencing data for estimating the abundance of intestinal immune cells in a given sample. In this study, we utilized the CIBERSORT algorithm within R software to calculate the proportions of 8 distinct immune cell types across various ET-infection samples present in GSE39602. Additionally, we visually depicted the composition of intestinal immune cells using box plots. Statistical significance was determined by conducting T-tests and variance tests to assess differences in immune cell proportions, with a significance threshold set at P < 0.05.

## 3 Results

### 3.1 DEGs in ET-infection and functional analysis

**Figure 1:**
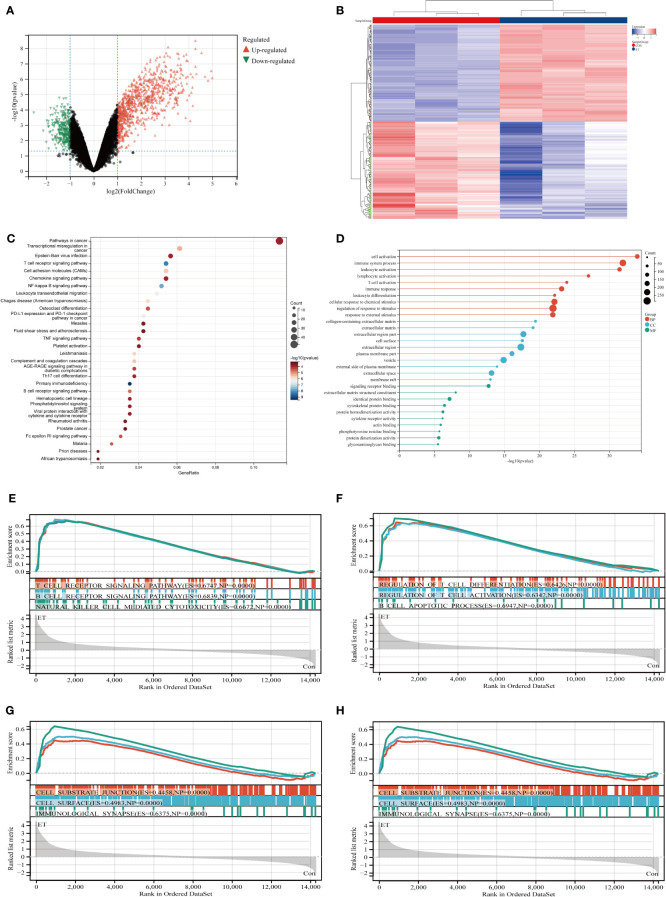
Identification and functional enrichment analysis of DEGs from GSE39602 dataset. **(A)** Volcanic maps of DEGs; **(B)** Heat maps of DEGs; **(C)** Top 30 KEGG pathways of DEGs; **(D)** Top 10 BP terms, CC terms, and MF terms of GO analysis in DEGs; **(E–H)** GSEA analysis of DEGs. CON, the control group; ET, *Eimeria tenella*; DEGs, differentially expressed genes.

The GSE39602 dataset was retrieved from the GEO database for analysis. A total of 1089 DEGs were identified, including 800 up-regulated genes and 289 down-regulated genes. These DEGs are visually depicted using volcano plots (**Figure 1A**) and heat maps (**Figure 1B**). The DEGs were found to be significantly enriched in immune related pathways including the T cell receptor signaling pathway, TH17 cell differentiation, B cell receptor signaling pathway, T cell activation, immune response and immune system process based on KEGG (**Figure 1C**) and GO (**Figure 1D**) analysis. To corroborate the findings of KEGG and GO analysis, GSEA analysis was performed on GSE39602, with a specific focus on immune-related signaling pathways encompassing T cell receptor signaling, B cell receptor signaling, natural killer cell-mediated cytotoxicity, and B cell apoptotic processes (**Figures 1E–H**).

### 3.2 Construction of WGCNA network in the GSE39602 dataset

**Figure 2:**
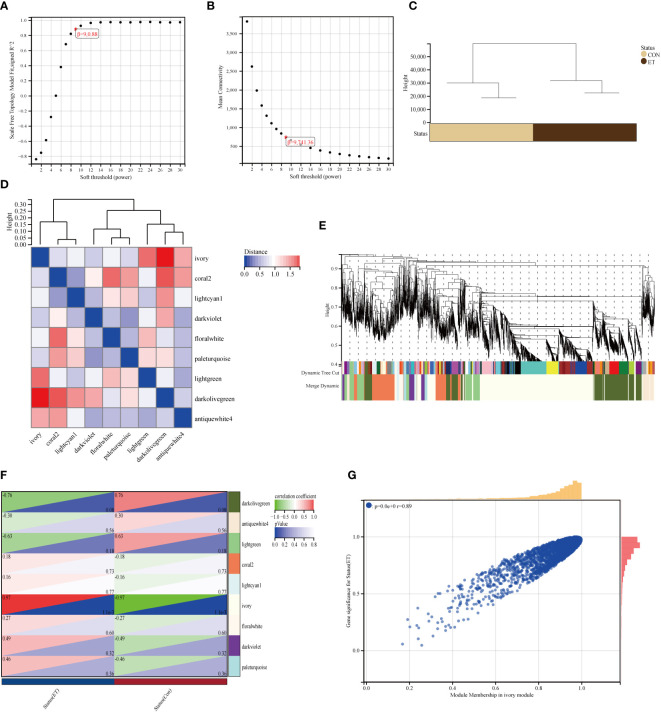
Construction of the expression network of ET-infection in the GSE39602 dataset. **(A, B)** The fitting index of network topology with scale-free characteristics is obtained by conducting soft threshold power analysis; **(C)** Sample clustering; **(D)** The heat map depicts the topological overlap matrix (TOM) of genes selected for weighted coexpression network analysis; **(E)** The coexpression clusters, along with their corresponding color allocation, are identified through hierarchical cluster analysis. Each color represents a distinct module within the gene co-expression network constructed using WGCNA; **(F)** The relationship of Groups and 9 modules; **(G)** The scatter plot demonstrates the correlation between MM and GS within the ivory module. CON, the control group; ET, *Eimeria tenella*.

The soft-power thresholds were determined based on the scale independence (**Figure 2A**) and mean connectivity (**Figure 2B**). Furthermore, it was observed that the samples displayed clustering behavior (**Figure 2C**). Additionally, a total of 9 co-expression modules were obtained by merging modules with distances less than 0.25. Moreover, module feature vector clustering (**Figure 2D**) and gene clustering (**Figure 2E**) were performed. According to the analysis results, a relatively high degree of independence is observed between modules in terms of gene expression. **Figure 2F** illustrates the analysis conducted on significant modules during ET-infection. We aimed to identify associations with the highest level of significance following ET-infection and module correlation. The findings revealed that module ivory exhibited the most substantial association with ET-infection (**Figure 2F**). Furthermore, there was a significant correlation between the mean value of ivory modules and gene significance (GS) (**Figure 2G**). We computed the correlation between the module feature vector and gene expression to derive the module membership (MM). By applying a cut-off criterion (|MM| > 0.8), we identified 2491 genes exhibiting high connectivity within the clinically significant module, thus designating them as module-hub genes.

### 3.3 Functional analysis of the ivory module

**Figure 3:**
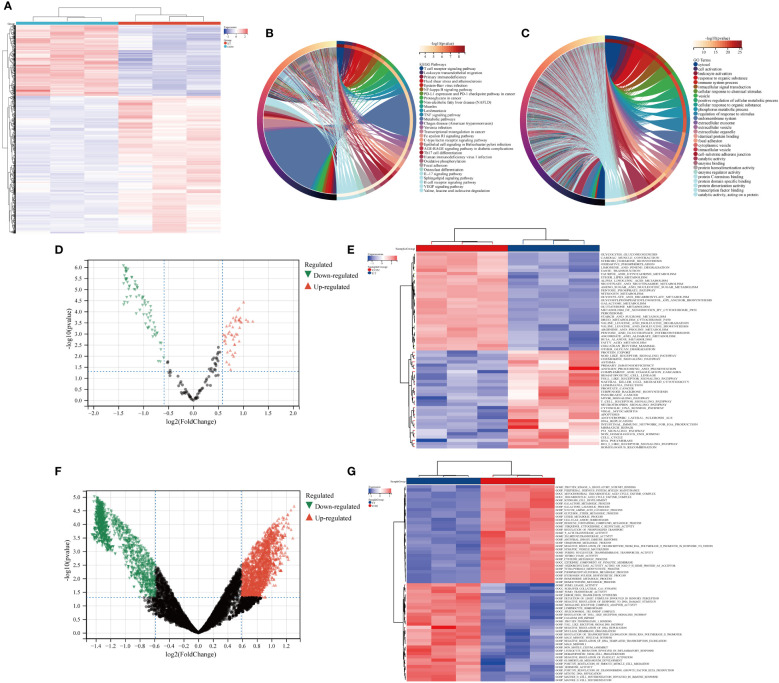
Functional enrichment analysis of ivory module genes. **(A)** Heat maps of ivory module genes; **(B)** Top 30 KEGG pathways of ivory module genes; **(C)** Top 30 GO analysis of ivory module genes; **(D, E)** GSVA analysis of ivory module genes based on KEGG gene sets; **(F, G)** GSVA analysis of ivory module genes based on GO gene sets. CON, the control group; ET, *Eimeria tenella*.

The heat map depicted the expression patterns of module-hub genes across different groups (**Figure 3A**). Furthermore, KEGG and GO analyses revealed that these module-hub genes were predominantly enriched in immune-related pathways, including the T cell receptor signaling pathway, TNF signaling pathway, Th17 cell differentiation, B cell receptor signaling pathway, cellular activation, immune system processes, and other relevant pathways (**Figures 3B, C**).

We further employed GSVA to analyze module-hub genes and validate the functional significance of the ivory module by assessing their enrichment in KEGG gene sets and GO gene sets. The volcano map and heat map revealed that within these gene sets, module-hub genes were predominantly enriched in crucial pathways such as T cell receptor signaling pathway, nod-like receptor signaling pathway, toll-like receptor signaling pathway, leukocyte migration involved in inflammatory response, mature B cell differentiation involved in immune response, and mature B cell differentiation (**Figures 3D–G**).

### 3.4 Identification and functional analysis of hub genes associated with CME and acquisition of biomarkers

**Figure 4:**
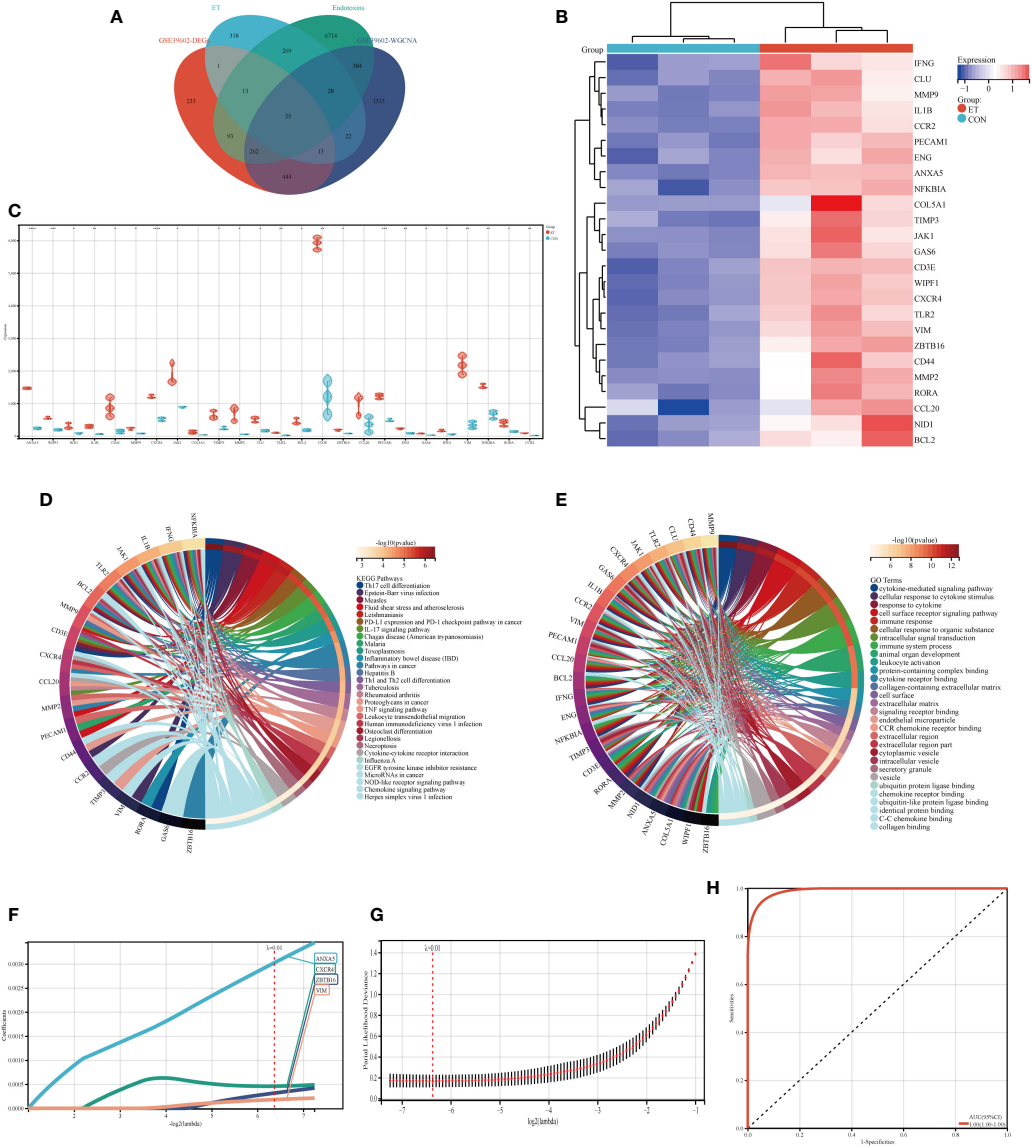
Identification of hub genes and potential biomarkers. **(A)** Intersection gene of DEGs, ET, Endotoxins, and ivory module gene; **(B)** Heat maps of hub genes; **(C)** The expression of hub genes in GSE39602; **(D)** Top 30 KEGG pathways of hub genes; **(E)** Top 30 GO analysis of hub genes; **(F, G)** Screening of potential biomarkers from hub genes using the LASSO algorithm; **(H)** Receiver operating characteristic (ROC) curve of predicted risk scores in ET-infection. CON, the control group; ET, *Eimeria tenella*; DEGs, differentially expressed genes. * P<0.05; ** P<0.01; *** P<0.005; **** P<0.001.

Venny diagram illustrates that the hub genes consist of a total of 25 genes (**Figure 4A**). The heat map demonstrates the expression levels of each individual hub gene across different groups (**Figure 4B**), while the box plot analysis reveals variations in expression for each hub gene (**Figure 4C**). The findings indicate significant differences in the expression patterns of all hub genes, except COL5A1 and CCL20, when compared to the control group. Moreover, KEGG and GO analyses revealed that the hub genes were predominantly enriched in pathways associated with Th17 cell differentiation, Th1 and Th2 cell differentiation, TNF signaling pathway, immune system processes, and immune responses (**Figures 4D, E**).

The LASSO algorithm was employed to identify four potential biomarkers, namely ANXA5, CXCR4, ZBTB16, and VIM, out of 25 hub genes that exert a significant impact on CME related ET-infection. The optimal model was constructed using the following formula: RiskScore=0.0030*ANXA5 + 0.0004*CXCR4 + 0.0003*ZBTB16 + 0.0001*VIM (**Figures 4F, G**). Furthermore, ROC analysis was utilized to evaluate the predictive performance of the constructed model, demonstrating its efficacy in outcome prediction (**Figure 4H**).

### 3.5 Construction of PPI network of DEGs and hub genes

**Figure 5:**
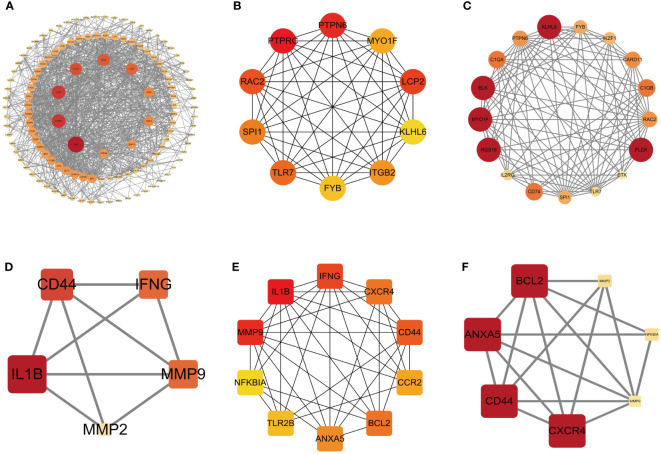
PPI network of DEGs and hub genes. **(A)** PPI network of DEGs based on Centiscape 2.2 plug-in. **(B)** Top 10 DEGs explored by CytoHubba plug-in. **(C)** PPI network of DEGs based on clustery analysis using the MCODE plug-in (score: 12.5). **(D)** PPI network of hub genes based on Centiscape 2.2 plug-in. **(E)** Top 10 hub genes explored by CytoHubba plug-in. **(F)** PPI network of hub genes based on clustery analysis using the MCODE plug-in (score: 5.667). The node color of Cluster was from pale yellow to red, and the corresponding score gradually larger. DEGs, differentially expressed genes.

To comprehensively investigate the role of proteins in bacterial endotoxin-related ET-infection, we conducted a comparative analysis of protein interactions between DEGs and hub genes using Centiscape 2.2, CytoHubba, and MCODE plug-ins. The results of the PPI network analysis revealed CD4, PTPRC, IL1B, PTPN6, LCP2, KLHL6, BLK, MYO1F, RGS18, and PLEK as identified hub DEGs (**Figures 5A–C**). Furthermore, the PPI network demonstrated that IL1B, ANXA5, CXCR4, MMP9, CD44, and BCL2 are crucial hub genes (**Figures 5D–F**). Moreover, combined with the results from the LASSO algorithm analysis, these findings highlight ANXA5 and CXCR4 as core potential biomarkers in CME related ET-infection.

### 3.6 Relationship between ET-infection and intestinal immune cells

**Figure 6:**
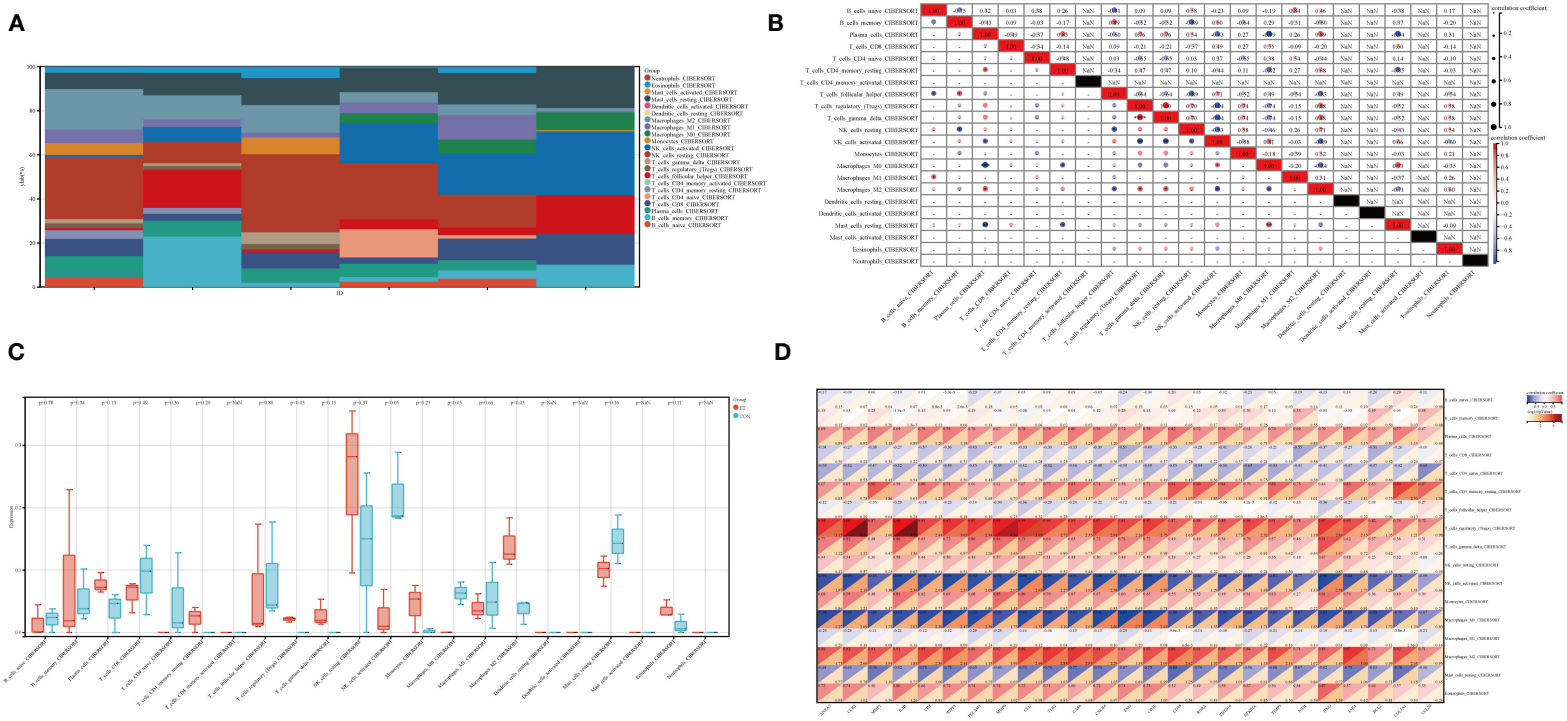
Immune characteristics in ET-infection. **(A)** The superposition diagram depicts the distribution of immune cell composition in the CON and ET groups, wherein each color corresponds to a distinct immune cell subtype. The horizontal axis represents different samples; **(B)** Correlation between different immune cells in ET-infection; **(C)** Expression variations among distinct immune cells in ET-infection; **(D)** The correlation between immune cells and hub genes in ET-infection. CON, the control group; ET, *Eimeria tenella*.

The immune tumor biology computational tool IOBR (Zeng et al., 2021) was employed in this study to analyze the expression profile and calculate the scores of eight immunoinfiltrating cell types in each sample using the CIBERSORT method (Newman et al., 2015), which was implemented through R software package IOBR. The present study investigated the infiltration and correlation of intestinal immune cells (**Figures 6A, B**), expression of intestinal immune cells between ET and CON groups (**Figure 6C**), as well as the association between intestinal immune cells and hub genes (**Figure 6D**). Spearman’s method was employed for visualizing the results of correlation analysis. The Treg cells are associated with both the T gamma delta cells and plasma cells, while the activated NK cells are linked to the resting NK cells, T gamma delta cells, Treg cells, and plasma cells (**Figure 6B**). Additionally, M0 macrophages are correlated with activated NK cells, T CD4 memory activated cells, and plasma cells (**Figure 6B**). Furthermore, there is a relationship between M0 macrophages and M0 macrophages as well as activated NK cells, T gamma delta cells, Treg cells and plasma cells (**Figure 6B**). Significant differences were observed between the ET and CON groups in terms of activated NK cells, M0 macrophages, M2 macrophages, and Treg cells,. Specifically, enhanced expression of Treg cells and M2 macrophages was noted in the ET group, while activated NK cells and M0 macrophages were suppressed (**Figure 6C**). Moreover, these four cell types exhibit a close association with the expression of hub genes, including CCR2, VIM, WIPF1, GLU, TLR2, CXCR4, and CD3E (-log10(P Value > 2) (**Figure 6D**). The core potential biomarker and the CME-related gene CXCR4 were ultimately identified, which is implicated in immune cell expression.

## 4 Discussion

The infection of ET can modify the diversity and composition of cecum flora. In comparison to the control group, there was a significant reduction in populations of *Proteobacteria*, *Enterococcus*, *Incertae*, *Escherichia–Shigella*, *Lactobacillus*, *Faecalibacterium*, and *Roseburia*. Conversely, populations of *Alistipes*, *Prevotella pectinovora*, *Clostridium*, *Lysinibacillus Escherichia*, *Bacteroidales*, and *Rikenella* increased significantly (Huang et al., 2018; Zhou et al., 2020; Yu et al., 2023). These findings indicate the presence of a diverse array of pathogenic bacteria in the upregulated flora during ET-infection. It is imperative to investigate the impact of up-regulation of pathogenic flora on ET-infection, and functional analysis of DEGs, module-hub genes, and hub genes revealed their significant enrichment in immune-related pathways. Therefore, this study further investigates the impact of caecal microbiome alterations on ET-infection by examining the correlation between CME-related genes and ET-infection as well as intestinal immune cells. We have identified a set of 25 CME related hub genes and subsequently determined that CXCR4 functions as a pivotal potential biomarker and immunotherapy target significantly associated with expression of intestinal immune cells. Therefore, we postulated that alterations in the caecal microbiome during ET-infection may lead to subsequent modulation of CXCR4 expression and immune cell activity, ultimately influencing the course of ET-infection.

The immune regulation of ET heavily relies on the induction of adaptive immunity through auxiliary Th1 responses and IFN-γ production (Hunter and Sibley, 2012). Furthermore, IL-17 production and its associated inflammation are also stimulated, in addition to promoting a Th1 response (Drinkall et al., 2017; Raballah et al., 2017). Whereas, the production of IL-17 appears to contribute to the pathological mechanisms associated with Apicomplexan infection (Ivanova et al., 2019). The immune response to infection of Apicomplexan involves the participation of diverse innate immune cell populations, including granulocytes, monocytes, macrophages, dendritic cells, and mast cells (Iwasaki and Akashi, 2007). These cellular components mount a reaction against infectious opportunistic lymphocyte populations through the production of chemokines and cytokines while presenting antigens. NK cells, classified as Group I innate lymphoid cells (ILCs), possess cytotoxic capabilities that distinguish them from other Group ILCs (Spits et al., 2013; Cortez and Colonna, 2016). The NK cells collaborate with the ILC2 and ILC3 cell populations to generate heightened levels of IFN-γ and TNF-α in response to IL-12, IL-15 and IL-18 stimulation induced by Th1 cells (Cortez and Colonna, 2016). The role of NK cells in ET-infection remains elusive. Previous studies have demonstrated that NK cells do not exert a protective role in Eimeria infection in mice (Smith et al., 1994). Moreover, following infection, the activity of NK cells in the spleen and intestine initially declines but subsequently recovers in chickens (Lillehoj, 1989), which may be associated with the observed decline of NK cells expression reported in this study. Furthermore, this study unveils a downregulation of NK cell expression during ET-infection, which is associated with the expression of CME-related genes including CCR2, VIM, WIPF1, GLU, TLR2, CXCR4 and CD3E. Consequently, it is postulated that perturbations in the intestinal microbiota caused by ET-infection may impede NK cell expression through the modulation of CME-related genes thereby facilitating ET-infection.

Treg cells, a subset of T cells involved in immunosuppression, comprise CD4+CD25+ T cells that suppressing activated immune cells in avian species (Shanmugasundaram and Selvaraj, 2011). Treg cells possess the capability to secrete a substantial quantity of IL-10, TGF-β, CTLA-4, and LAG-3 (Selvaraj, 2013), with particular emphasis on the pivotal role played by IL-10 in evading the host immune response. Treg cells possess the capacity to suppress Th17 cells, thereby attenuating tissue damage caused by ET-infection (Kim et al., 2019b). However, an alternative hypothesis suggests that coccidial parasites have evolved to induce IL-10 expression in Treg cells, promoting chicken invasion and survival through inhibition of the IFN-γ-associated Th1 response (Kim et al., 2019a). Therefore, it is essential to investigate the role of Treg cells in the pathogenesis of ET-infection. The findings in this study demonstrate that Treg cells are also upregulated during ET-infection, which correlates with the differential expression of numerous CME-related genes in these cells. We propose that during ET-infection, changes in the cecal microbiota induce endotoxin-mediated activation of Treg cells, leading to their upregulation. Consequently, this may result in an elevation of IL-10 expression, thereby suppressing host immunity and facilitating ET-infection.

The occurrence of acute inflammation is the primary etiological factor underlying cecum injury subsequent to ET-infection, and this process is characterized by the infiltration of inflammatory cells and the release of pro-inflammatory mediators, including IFN-γ, IL-1β, IL-6, and IL-17 (Laurent et al., 2001; Hong et al., 2006). Macrophages, as components of the innate immune system, are capable of inducing the production of IL-1β, IL-6, and IL-17, in addition to various chemokines subsequent to ET-infection. This process facilitates the initiation and progression of inflammation. Alterations in the cecal microbiota have been demonstrated to play a pivotal role in macrophage recruitment. However, ET-infection alone is insufficient for fulfilling this function. Moreover, the microbiota can stimulate the expression of pro-inflammatory genes, thereby contributing to the development of cecal lesions (Tomal et al., 2023b). The presence of endotoxins and IFN-γ has been demonstrated to induce the polarization of M0 macrophages towards the M1 phenotype, while IL-4 and IL-13 drive the polarization of M1 macrophages into the M2 macrophages (Popena et al., 2018). Moreover, M2 macrophages possess the capability to secrete pro-inflammatory cytokines such as IL-1β and TNF-α, which contribute to the progression of inflammation (Popena et al., 2018). It is noteworthy that in this study, we observed an association between CME-associated genes and the downregulation of M0 macrophages as well as the upregulation of M2 macrophages. Therefore, our hypothesis posits that CME may potentially exacerbate inflammation by modulating the polarization of M0 macrophages towards an M2 macrophages, thereby facilitating ET-infection.

In summary, based on bioinformatics, this study revealed that CXCR4 associated with CME exert a regulatory influence on the activity of NK cells, M0 macrophages, M2 macrophages, and Treg cells during ET-infection, thereby impacting the progression of ET-infection. Therefore, CXCR4 is expected to emerge as a potential biomarker and immunotherapy target for elucidating the impact of caecal microbiome on ET-infection. These findings provide valuable insights into the involvement of cecal flora in ET-infection.

## Data availability statement

The datasets presented in this study can be found in online repositories. The names of the repository/repositories and accession number(s) can be found in the article/supplementary material.

## Author contributions

MH: Data curation, Methodology, Software, Visualization, Writing – original draft, Writing – review & editing. JL: Resources, Software, Writing – original draft, Writing – review & editing. YW: Methodology, Validation, Writing – original draft. JzL: Data curation, Funding acquisition, Resources, Supervision, Validation, Writing – review & editing.
